# The Hospitals Association

**Published:** 1896-05-02

**Authors:** 


### THE HOSPITALS ASSOCIATION.

### A CENTRAL HOSPITALS BOARD.

A largely-attended meeting of the Hospitals Association was held at the Board-room of the Charing Cross Hospital on the 23rd ult., under the chairmanship of Lord Monkswell (a member of the late Select Committee of the House of Lords on Hospitals), to hear a paper by the Hon. Sydney Holland, on "A Central Body for the Supervision of London Hospitals." There were about 150persons present, including representatives of most of the large metropolitan hospitals, the Hospital Sunday and Saturday Funds, the Charity Organisation Society, &c.

### Mr. Holland's Paper.

The Hon. Sydney Holland, in the course of his paper, said that he had come to the conclusion, from talking with hospital authorities, with the subscribers to many hospitals, and with many medical men, that a central board was needed. There were, however, difficulties in the way—first, "self-righteousness," *i.e.*, the belief which existed in the minds of almost everybody connected with every hospital that their hospital was perfect; and 'secondly, the supposed danger that a central body would tend to lessen the individual interest of those who are now managing the different hospitals. In the latter case how could the existence of a central body lessen the keenness of anyone who was really anxious that his hospital should be perfect? It might dull that keenness if his responsibility in the management of his hospital was to be shared, but the existence of a central board could never dull his keenness if, as would be the case, it was found that the result of the good work was to have that work recognised, pointed out and followed by others, whilst the result of bad work was not only to get blame but to lose money in consequence. (Hear, hear.) It was objected that a central body would lessen the amount given to hospitals generally. He did not believe it would. Confidence had never yet lessened the circulation of money, and he believed that blessings would be showered—followed by cheques—on any body of men who would exercise such control over hospitals that people who wished to subscribe could feel sure that only hospitals managed up to a certain pitch of excellence, and built where they were needed, would participate. (Applause.) Nor did he think a central body would lessen the amount subscribed to individual hospitals, as most donations and subscriptions were generally given to individual hospitals for personal reasons. After showing what duties they should undertake, Mr. Holland went on to discuss the composition of the central board. One important matter had to be kept in view, and that was that the board must not be a new one. Some of the reasons for this were (1) that it would have to be in existence years before it could gain the confidence of the public; (2) it would have no money to carry on its organisation; (3) before it could do any good work, internecine strife would probably have brought it into disrepute, and parties within parties have been formed; and when this excellent body had got rid of its monthly (4) even nurse, and could sit up, who would pay attention to its howls, except to vote it a nuisance? It would have no power. To give a central board power it must be a body now known, now popular, and above all things, it must command the purse strings. Inspection by however worthy a body, without any power of enforcing its remarks, would be useless. People were sick of reports by retired officials and by experts in theories—but if the central board were able to say "If this be not remedied we can give no grant," such an argument would come home at once, even to the most ignorant and sleepy of hospital committee men. The correct view of hospital workers should be this: "We recognise the right of our own subscribers, *i.e.*, those who support us, to ask for a central board, we recognise the right of the medical profession to ask for it, and we who are managing hospitals are quite willing to join in and help a properly constituted board to be an efficient, useful, and representative body. (Hear, hear.) But we will not silently agree to any outside body being forced upon us by people who do not subscribe to or help us. (Hear, hear.) We look with suspicion, not to say hatred, on faddists and fussy people, who readily undertake to manage anybody and anything with happy ignorance of all detail, and who are always ready with their advice or a letter to a newspaper." A body which fulfilled all the conditions he had laid down was the Hospital Sunday Fund Council. (Hear, hear.) It was already in power, was well known, exceedingly popular, trusted by the public, and was a body of certain continuance. (Hear, hear.) But they must insure that there were on that council a sufficient number of representative hospital men to lessen the danger of the clerical element becoming too strong where the work and responsibility were to be so largely increased. Therefore the Hospital Sunday Fund Council should consist, as at present, of a number of contributors to the fund, an increased number of representatives of hospitals, and also of representatives of the medical and nursing professions. Then, having a council so formed, they should elect from themselves an executive central hospital board in the form of a distribution committee, consisting of, say, fifteen members, *i.e.*, five laymen, five hospital experts, four medical representatives, and one nursing representative, and it would be well that the hospital representatives on the Council should elect their own representatives on the distribution committee, the medical men theirs, and so on, so as to ensure the very best men being on the distribution committee. This distribution committee should select from amongst the members of the council a visiting committee of, say, 30, composed of experts, who should divide up into sub-committees of not less than two members, and in the course of the year visit every hospital to which funds were given, or which sent in a demand to share in the fund. The visiting committee would report to the distribution committee, who would, having considered this report, state their reasons for withholding or increasing any grant over and above the normal ratio arrived at as at present. After meeting possible criticisms of the scheme, Mr. Holland concluded his paper amid much applause. He then moved the following resolutions:—

1. That a central body for the supervision of London hospitals is needed, and the appointment of it should no longer be delayed.

2. That it is desirable to reconstitute the Hospital Sunday Fund Distribution Committee by the addition of representatives of the London hospitals, so as to form the central body representative of those associations.

### The Discussion.

The Chairman, in opening the discussion, said he thought Mr. Holland carried the audience with him when he said it was necessary to have a central board. He thought, however, that Mr. Holland was a little misleading in his proposal as to the constitution of the board. Mr. Holland said that it was essential there should not be a new board, but to his humble intelligence it appeared that the reconstituted Hospital Sunday Fund Council was a new board, and that the objections to having a new board militated equally against the reconstructed council. Mr. Holland's paper was also not quite satisfactory as to what the power of the central board should be. The board, as outlined, was only to "suggest" that certain things should be done, but it seemed to him that the distinction between having the power to suggest but not having the power to interfere in the management of hospitals was rather thin, and he was not therefore certain what was the exact position Mr. Holland intended his central board to occupy. After detailing what the suggestions of the Lords' Committee were, the Chairman said he thought a good deal might be said against the proposition that the same body should deal with the distribution of the funds as dealt with suggestions as to management. His opinion was that the examining body and the schoolmaster body should not be one and the same.

It was decided, after a brief discussion, that resolution No. 1 should be first put before the meeting.

Mr. R. B. D. Acland (chairman of the Hospital Saturday Fund) seconded the resolution.

The Chairman was about to put the resolution to the meeting, when

Mr. T. Ryan (secretary of St. Mary's Hospital) interposed. He regretted that he, a paid official of a hospital, should have to interfere at that stage of the proceedings, but he feared that that resolution might have been put and carried as really representing the general opinion of that meeting. He thought it would be generally felt by the representatives of hospitals present that the better way to act would be to call the accredited representatives of hospitals together in council and obtain their views before adopting a resolution such as that proposed. He did not oppose the spirit of the resolution—he, in fact, highly approved of a central board being appointed—but he entertained the strongest opinion that the adoption of that body by the hospitals depended upon the position taken up initially.

The Chairman thought that Mr. Ryan's objection would come better under the second resolution.

Mr. Ryan did not think so, as, in his opinion, the point he had raised was of vital importance to the carrying out of a central board at all. He moved, as an amendment—

> That it is desirable to ascertain the opinion of the governing bodies of the London hospitals as to the desirability of appointing a central board for the supervision of London hospitals. (Applause.)

He did not move this amendment in any sense of opposition to the principle of the central board. His feeling was that if this board was to be useful and to do what it should do it should be self-imposed by the hospitals on themselves, for themselves, and for their own improvement. (Hear, hear.)

Dr. Gilbart Smith seconded, as he thought Mr. Ryan had taken the wisest course, because Mr. Holland's proposition was, as the chairman had shown them, really unworkable; at any rate, the chairman had shown that the body proposed was a new board.

The Chairman: Surely this is rather out of order.

Dr. Gilbart Smith: At any rate, I object to voting on this resolution until we have decided what the body will be. We are in the frying-pan now, but we may be in the fire if the resolution as proposed is carried. (Laughter.)

Lord Knutsford did not think Dr. Gilbart Smith's speech was to the point. This question of a central board did not come up now for the first time, as there had been a Royal Commission on the subject, and the matter had been discussed more than once. They would never get on if they had again to go back to the hospital authorities. That meeting had a perfect right, without referring again to the hospital authorities, to carry the resolution proposed by Mr. Holland.

Mr. Harrold (secretary of the Royal Hospital for Diseases of the Chest) said it was not only the mere fact that the subscribers to hospitals had taken but little interest in the matter, but the managing bodies—his Council at any rate—had never had the question put before them in anything like a definite shape. The mere fact that the Charity Organisation Society had called a meeting, and that the present meeting had been held, did not impress the committees of hospitals as being anything very serious or terrible, therefore they had not taken very much notice of the matter.

Mr. Thornton Sharp (Golden Square Throat Hospital) did not see the necessity for a central board. He objected to it because no guarantees had been given them that a central board would know more about hospital management than the managers of the hospitals did. Personally, he would rather have rate-supported hospitals than a central board, elected by nobody apparently. They, the managers of hospitals, were after all put in their places by the people who found the funds, and those people were satisfied with the management.

Mr. Timothy Holmes, F.R.C.S., believed the hospitals, using that word to represent the larger and better-known hospitals, would be exceedingly glad to welcome a central board—(hear, hear)—always supposing that central board was properly constituted. Mr. Ryan's amendment seemed like putting the cart before the horse. It was perfectly useless to consult the hospitals about whether they wished for a central board or not until they decided what that central board was to be.

Mr. St. Vincent Mercier (secretary of the St. John's Hospital for Diseases of the Skin), who spoke amid much disturbance, thought the subscribers to hospitals should be consulted before that meeting decided that a central board was necessary.

Mr. Loch (secretary to the Charity Organization Society) suggested, in order that the issue before the meeting might not be confused, that Mr. Ryan should add his amendment as a rider to the resolution.

Mr. Ryan accepted this suggestion.

Mr. Jennings (secretary to the Chelsea Hospital for Women) thought that before passing either resolution or amendment, the constitution of the central board should first be considered.

Mr. B. Burford Rawlings (secretary to the National Hospital for the Paralysed and Epileptic) thought the passing of the resolution would affirm that those present, as managers of hospitals, considered that the hospitals were in need of supervision. That was exactly what the Charity Organization Society had been so persistently trying to get them to say in the past, and he strongly deprecated their doing so in any way.

Mr. R. B. D. Acland said it was a notable fact that no single person had yet spoken against the resolution except those gentlemen (*i.e.*, the paid officials) who were interested—he did not say so unkindly, but it was so—in the maintenance of the present state of affairs. (Cries of "No, no!")

Mr. Thornton Sharp: I am not a paid official of any hospital. (Hear, hear.)

Mr. Acland: Then I retract as regards Mr. Sharp. Continuing he said that all the discussion that evening had been from the point of view only of the boards of management. Nothing had been said of the interests of the patients or the servants.

Dr. Gilbart Smith: When I alluded to the fact that it was possible our hospitals might be in the fire I meant the hospital from the patients' point of view.

Mr. Acland: I only regret Dr. Gilbart Smith did not express himself more clearly. Looking at the question rather as it affected the sick poor throughout London, than as it affected individual hospitals, Mr. Acland desired to put on record his firm conviction that many of the failures and defects which they saw at the present time could not possibly be cured without the institution, by some means or another, either voluntarily or compulsorily, of some central board of management. There was no idea of interfering with the internal management of hospitals, but they were anxious to help the hospitals to solve the greater problems presented by the question of medical relief in London.

Dr. Allchin objected to Mr. Acland's reference to all those opposed to the scheme being merely paid officials. His experience led him to believe that the formation of a central board was not desirable.

Sir Savile Crossley, Bart. (Hospital Sunday Fund Distribution Committee), thought both the managers and the subscribers of hospitals had had plenty of opportunity of discussing that question. He should vote for the resolution.

The Chairman, reading the resolution, said that, as Mr. Ryan had consented to add his amendment as a rider, it would, perhaps, be best to first put the resolution, and then take the sense of the meeting as to whether the rider should be added or not.

Dr. Gilbart Smith said that as seconder he would prefer that the amendment should not be added as a rider. This question was one which had never before been considered by the association.

The Chairman: I beg your pardon. I have seen a letter from the council saying that the Hospitals Association are in favour of it.

Dr. Gilbart Smith did not remember that the question had ever been put before the association before. In his view it was only courteous to the hospitals themselves, before deciding on the main question, to ask what their opinion was.

Mr. Ryan: I am ready to defer to Dr. Gilbart Smith's wishes, and to leave the amendment as I originally moved it.

The Chairman: Those who vote for the amendment will vote for burking the question at present. (Cries of "No, no," "Yes, yes.") Well, it means, Mr. Ryan, that this meeting refuses to express any opinion.

Mr. Ryan: Yes.

The Chairman: Well, I call that burking the question. If the amendment is carried there can be no further expression of opinion as to whether a central board is desirable or not.

The Chairman then put the amendment, which was carried by 21 votes to 19.

Votes of thanks to Mr. Holland (proposed by Colonel Montefiore, and seconded by Mr. Ryan), and to the Chairman, brought the proceedings to a close.

